# Association between hypotension and serious illness in the emergency department: an observational study

**DOI:** 10.1136/archdischild-2018-316231

**Published:** 2019-04-04

**Authors:** Nienke N Hagedoorn, Joany M Zachariasse, Henriette A Moll

**Affiliations:** Department of Pediatrics, Erasmus MC-Sophia, Rotterdam, The Netherlands

**Keywords:** hypotension, emergency medical service, predictive value, vital signs, serious illness

## Abstract

**Background:**

The value of routine blood pressure measurement in the emergency department (ED) is unclear.

**Objective:**

To determine the association between hypotension in addition to tachycardia and the Shock Index for serious illness.

**Design:**

Observational study.

**Setting:**

University ED (2009–2016).

**Participants, methods and main outcomes:**

Routine data collected from consecutive children <16 years. Using logistic regression, we assessed the association between hypotension (adjusted for tachycardia) and Shock Index (ratio heart rate/blood pressure [BP]) for serious illness. The predictive accuracy (sensitivity, specificity) for hypotension and Shock Index was determined for serious illness, defined as intensive care unit (ICU) and hospital admissions.

**Results:**

We included 10 698 children with measured BP. According to three age-adjusted clinical cut-offs (Advanced Paediatric Life Support, Paediatric Advanced Life Support and Paediatric Early Warning Score), hypotension was significantly associated with ICU admission when adjusted for tachycardia (range OR 2.6–5.3). Hypotension showed low sensitivity (range 0.05–0.12) and high specificity (range 0.95–0.99) for ICU admission. Combining hypotension and tachycardia did not change the predictive value for ICU admission. Similar results were found for hospitalisation. Shock index was associated with serious illness. However, no specific cut-off value was identified in different age groups.

**Conclusions:**

Hypotension, adjusted for tachycardia, is associated with serious illness, although its sensitivity is limited. Shock index showed an association with serious illness, but no acceptable cut-off value could be identified. Routine BP measurement in all children to detect hypotension has limited value in the ED. Future studies need to confirm which patients could benefit from BP measurement.

**What is already known on this topic?**

- Hypotension is considered a late feature of serious illness in children and different reference values exist for hypotension.
- In the adult emergency department population, high Shock Index is associated with mortality, severity of illness and hospital admission.

**What this study adds?**

- Hypotension has additional value over tachycardia, but due to its low sensitivity clinical relevance is limited.
- High Shock Index is associated with serious illness in different age groups. Acceptable cut-off values could not be identified.
- Blood pressure measurement for detection of hypotension is suggested to be of limited value in all children attending the emergency department.

## Introduction

Vital signs are essential for recognising serious illness in children in the emergency department (ED). However, the frequency of blood pressure (BP) measurement varies widely (23%–87%) and no consensus exists on performing routine BP measurement to detect hypotension.^1–3^ Accurate age-related cut-offs are needed to assess hypotension as incorrect cut-offs may lead to false-positive or false-negative results. Although paediatric guidelines provide different definitions of low BP, it is unclear which BP cut-off should be used in the ED.^4–6^

Moreover, the predictive value of hypotension for serious illness is unclear in the diverse ED population. In children, hypotension is considered a late sign of deterioration and is used for diagnosis of shock. Children increase heart rate to preserve cardiac output.^7 8^ Since abnormal heart rate occurs in an earlier phase, the additional value of routine BP over heart rate in prediction of serious illness could be limited in the ED.

Another measure of haemodynamic status is Shock Index, the ratio of heart rate to systolic BP, which is associated with mortality and disease severity in adults.^9–11^ In small cohorts of children, elevated Shock Index has been associated with injury severity in trauma and mortality in septic shock.^12–15^ However, the Shock Index in all paediatric ED patients has not yet been evaluated and could be an important predictor in children.

This study aims to study the additional value of BP measurement: (1) To determine the predictive capability of hypotension in addition to tachycardia. (2) To assess the utility of Shock Index for serious illness in children. This observational study is based on routine BP measurements in the ED using electronic health records.

## Methods

### Design

We applied three commonly used clinical definitions for hypotension on data from a prospective study of children visiting the ED to determine the predictive value of hypotension in addition to tachycardia for serious illness. Second, we studied the predictive ability of the Shock Index. This was a secondary analysis in a study validating the Manchester Triage System (MTS).^16 17^

### Setting

The observational study included all children <16 years who presented consecutively at the ED of Erasmus MC-Sophia Children’s Hospital (Rotterdam, The Netherlands) between August 2009 and December 2016. This inner-city university hospital receives approximately 7000 children annually.

### Data collection

Data of patient characteristics, vital signs, triage level and disposition were automatically derived from electronic health records that were completed by trained nurses during triage. Heart rate was measured using pulse oximeters and BP using the oscillometric infinity M540 monitor (Draeger Medical, Telford, Pennsylvania, USA). BP was measured on medical indication at the discretion of the nurse or attending physician.

### Outcomes and definitions

Serious illness was defined as admission to the ICU or hospital following ED visit. Indications for ICU admission include requirement of advanced respiratory support ([non-] invasive ventilation, high flow oxygen); inotropes or continuous intravenous antiepileptics; tracheal cannula; acute or threatening failure of more than two organ systems which was expected to last >24 hours or in a child <1 year.^17^ We selected three age-adjusted clinical cut-offs to define hypotension to demonstrate the range in clinical practice: Advanced Paediatric Life Support (APLS),^18^ Paediatric Advanced Life Support (PALS)/septic shock screening tool^19 20^ and the Paediatric Early Warning Score^21^ (table 1). Heart rate was categorised as tachycardia versus no tachycardia according to the same reference as the BP cut-off (online supplementary appendix 1). Children with bradycardia (5.9%–7.4%)%) were defined as no tachycardia. Age was categorised as 0–1 year, 1–2 years, 2–5 years, 5–12 years and 12–16 years. Triage urgency was determined by MTS V.3.^22^ Ill appearance was assessed by the nurse on a 2-point scale: ill versus non-ill appearance.

10.1136/archdischild-2018-316231.supp1 Supplementary data

**Table 1:** Definition of hypotension in different age groups for systolic blood pressure in mm Hg

Age range	APLS^18^	PEWS^34^	PALS^19 20^
<4 weeks	<75	≤60	<60
4–6 weeks	<75	≤60	<70
6 weeks to 3 months	<75	≤60	<70
3–6 months	<75	≤80	<70
6–12 months	<75	≤80	<70
1–2 years	<75	≤90	<72
2–3 years	<80	≤90	<74
3–4 years	<80	≤90	<76
4–5 years	<80	≤90	<78
5–6 years	<90	≤90	<80
6–7 years	<90	≤90	<82
7–8 years	<90	≤90	<84
8–9 years	<90	≤90	<86
9–10 years	<90	≤90	<88
10–12 years	<90	≤90	<90
12–13 years	<105	≤100	<90
13–14 years	<105	≤100	<90
14–16 years	<105	≤100	<90

APLS, Advanced Paediatric Life Support; PALS, Paediatric Advanced Life Support; PEWS, Paediatric Early Warning Score.

### Data analysis

Our sample was limited to patients with measured heart rate and BP. Children who died in the ED were excluded (n=34). The value of BP measurement could be limited in this group, since the majority (94%) was triaged as emergencies. Outliers were verified in patient records. First, we assessed the relation between BP and heart rate, using scatter plots. To facilitate analysis across age groups, we standardised heart rate and BP using z-scores, which were calculated separately for the different age categories. Second, we assessed the association between hypotension and serious illness using the three clinical cut-offs for hypotension. We used univariable logistic regression to evaluate the association of different BP cut-offs with ICU or with hospital admission, and adjusted for tachycardia in a multivariable model.

We determined the predictive value of hypotension for ICU admission and hospitalisation by calculating sensitivity, specificity, and positive and negative likelihood ratios.^23^ To study the predictive value of hypotension in addition to tachycardia, we calculated the predictive value of (1) hypotension; (2) tachycardia; (3) the combination of tachycardia and hypotension; 4) Either hypotension or tachycardia. Positive likelihood ratios >5 and negative likelihood ratios <0.2 were considered relevant.^24^

The normal range of Shock Index (ratio of heart rate to BP) is age dependent.^25^ Therefore, we stratified the analysis for Shock Index by age. To assess the association of Shock Index, we used univariable logistic regression. To facilitate interpretation, the OR present the odds for 0.1 unit increase in Shock Index. Next, the discriminative ability was presented by the area under the curve (AUC) of receiver operating characteristics. We used Youden’s Index to identify the optimal cut-off value to assess the predictive value.^26^ We merged the age groups into <2 years, 2–10 years and >10 years to ensure sufficient numbers for statistical analysis. To explore age-adjusted cut-off values for high Shock Index, we defined a cut-off by dividing the APLS tachycardia value with the APLS hypotension value for each age group (online supplementary appendix 2).

Subgroup analyses were performed in patients with ill appearance, fever (temperature >38°C) and patients presenting with surgical problems including major trauma, head injury, limb problems, wounds, torso injuries and assault.^27^

Data analyses and visualisation were performed in SPSS V.24.0 and R. The medical ethical committee waived the requirement for informed consent.

## Results

During the study period, 45 495 children (58.6% male) presented to the ED; 891 (2.0%) were triaged as emergencies. A total of 10 698 patients had BP and heart rate measured. In this sample, 3907 (36.5%) children were admitted to the general ward and 631 (5.9%) were admitted to the ICU (table 2). Patients with BP measurement were older, had higher urgency level and were more often admitted compared with children without BP measurement (online supplementary appendix 3). The prevalence of hypotension ranged from 1.2% to 5.3% depending on the cut-off used (online supplementary appendix 4). In children with hypotension according to APLS, 13.9% were admitted to the ICU and 33.5% were hospitalised.

**Table 2:** Characteristics of visits at the paediatric emergency department of Sophia Children’s Hospital from 2009 to 2016

	Total	Patients with blood pressure and heart rate measured	Patients with hypotension according to APLS
	**n=**45 495	**n=**10 698	**n=504**
**Male; n %**	26 338 (57.9)	5872 (54.9)	219 (43.5)
**Age in years; median, (IQR)**	4.3 (1.4–9.8)	7.74 (3.6–7.7)	13.0 (6.67–14.5)
**Age category; n (%)**			
0–1 year	8734 (19.2)	920 (8.6)	78 (15.5)
1–2 years	5736 (12.6)	668 (6.2)	7 (1.4)
2–5 years	10 154 (22.3)	2091 (19.5)	14 (2.8)
5–12 years	13 503 (29.7)	4101 (38.3)	80 (15.9)
12–16 years	7368 (16.2)	2918 (27.3)	325 (64.5)
**MTS urgency; n (%)**			
Emergent/very urgent	6433 (14.2)	2572 (24.0)	155 (30.7)
Urgent	19 873 (43.7)	5026 (47.0)	199 (39.5)
Standard/non-urgent	17 711 (38.9)	2922 (27.3)	163 (27.0)
Missing	1478 (3.2)	178 (1.7)	14 (2.8)
**Disposition; n (%)**			
Admission general ward	8848 (19.4)	3276 (30.6)	169 (33.5)
Intensive care	1132 (2.5)	631 (5.9)	70 (13.9)
Died	34 (0.1)	–*	–*
Discharge	34 913 (76.7)	6719 (62.8)	261 (51.8)
Other	401 (0.9)	61 (0.6)	4 (0.8)
Missing	167 (0.4)	11 (0.1)	0 (0.0)
**Shock Index; mean (SD)**			
0–1 year		1.52 (0.48)	
1–2 years		1.25 (0.31)	
2–5 years		1.11 (0.26)	
5–12 years		0.89 (0.24)	
12–16 years		0.76 (0.22)	

*Children who died were excluded.

APLS, Advanced Paediatric Life Support; MTS, Manchester Triage System.

**Figure 1:**
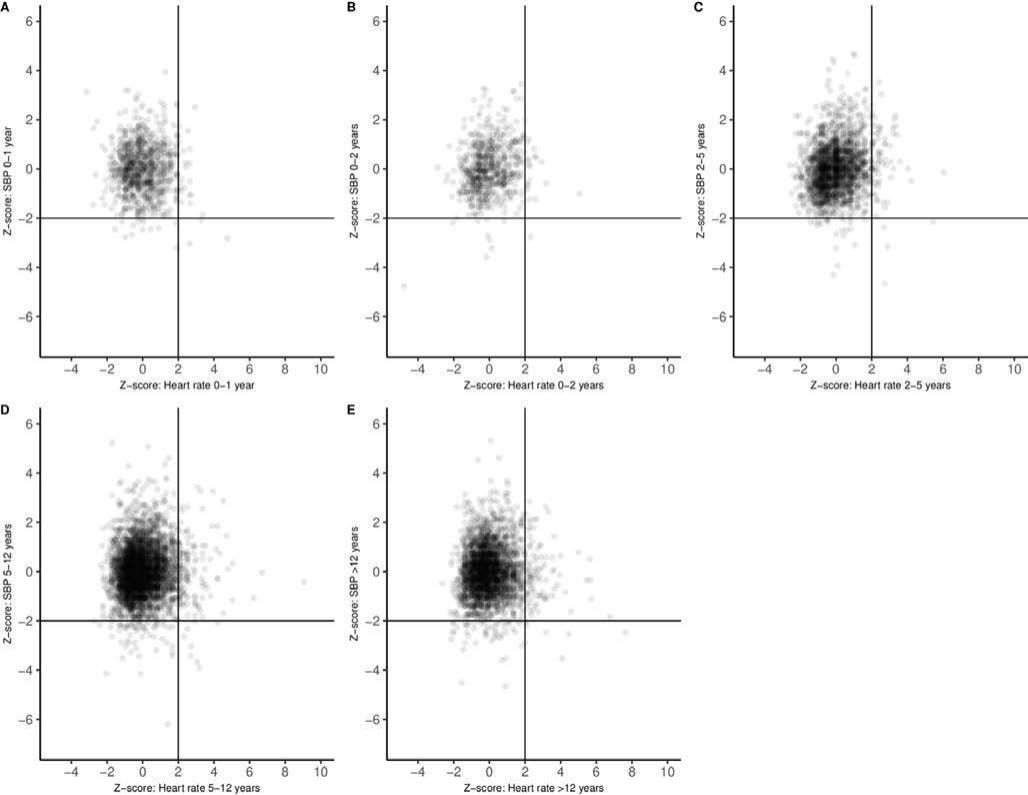
Scatter plots of z-scores of heart rate and systolic blood pressure (SBP) for different age categories (A; 0–1 year, B; 1–2 years, C; 2–5 years, D; 5–12 years, E; 12–16 years).

Our study found no association between z-scores of heart rate and BP in any of the age categories (Pearson correlation 0.04–0.18) (figure 1). In particular, no clear relation was observed between low BP and high z-scores for heart rate.

Hypotension, as a sole predictor, had an association with ICU admission (range OR 2.56–5.27) and hospital admission (range OR 1.4.66–2.66). The association between hypotension and serious illness remained significant after adjustment for tachycardia. In this analysis, the PALS cut-off for hypotension showed the strongest association with ICU admission and hospitalisation (table 3).

**Table 3:** Logistic regression analysis for ICU and hospital admission

	Patients with hypotension/tachycardia	ICU admission		Hospital admission	
		OR	95% CI	OR	95% CI
APLS^18^					
Hypotension	n=504	2.77	2.12 to 3.62	1.61	1.34 to 1.92
Tachycardia (APLS)	n=1692	2.46	2.06 to 2.94	2.62	2.36 to 2.91
Hypotension adjusted for tachycardia		2.68	2.05 to 3.51	1.56	1.30 to 1.88
PALS/septic shock screening tool^19 20^					
Hypotension	n=133	5.27	3.51 to 7.91	2.66	1.87 to 3.77
Tachycardia (septic shock screening tool)	n=1709	1.80	1.49 to 2.18	1.91	1.72 to 2.12
Hypotension adjusted for tachycardia		4.99	3.32 to 7.52	2.52	1.77 to 3.59
PEWS^34^					
Hypotension	n=571	2.56	1.98 to 3.31	1.46	1.24 to 1.73
Tachycardia (PEWS)	n=4113	2.02	1.72 to 2.37	2.16	1.99 to 2.34
Hypotension adjusted for tachycardia		2.54	1.96 to 3.29	1.46	1.23 to 1.73
Shock Index*					
Age 0–1 year		1.09	1.06 to 1.14	1.14	1.09 to 1.18
Age 1–2 years		1.07	0.99 to 1.16	1.07	1.02 to 1.22
Age 2–5 years		1.08	1.02 to 1.15	1.06	1.02 to 1.09
Age 5–12 years		1.13	1.08 to 1.19	1.14	1.11 to 1.18
Age >12 years		1.22	1.15 to 1.29	1.19	1.15 to 1.24

*ORs present each 0.1 increase in Shock Index.

APLS, Advanced Paediatric Life Support; ICU, intensive care unit; PALS, Paediatric Advanced Life Support; PEWS, Paediatric Early Warning Score.

The cut-offs for hypotension showed a low sensitivity and a high specificity for serious illness (table 4). For ICU admission, specificity ranged between 0.95 and 0.99 and sensitivity between 0.05 and 0.12. The positive likelihood ratios ranged from 2.38 to 5.06 and the negative likelihood ratios ranged from 0.93 to 0.96. The combination of tachycardia and hypotension did not improve the performance for ICU admission with low sensitivity (0.02–0.08) and high specificity (0.94–0.98). The analysis for hospital admission showed similar results.

**Table 4:** Predictive value for different cut-offs of hypotension and/or tachycardia for ICU admission and hospital admission

	ICU admission				Hospital admission			
	Sensitivity (95% CI)	Specificity (95% CI)	Positive likelihood ratio (95% CI)	Negative likelihood ratio (95% CI)	Sensitivity (95% CI)	Specificity (95% CI)	Positive likelihood ratio (95% CI)	Negative likelihood ratio (95% CI)
**Predictive value for hypotension**								
APLS	0.11 (0.09 to 0.14)	0.96 (0.95 to 0.96)	2.57 (2.03 to 3.27)	0.93 (0.90 to 0.96)	0.06 (0.05 to 0.07)	0.96 (0.96 to 0.97)	1.57 (1.32 to 1.86)	0.98 (0.97 to 0.99)
PALS/sepsis	0.05 (0.04 to 0.07)	0.99 (0.99 to 0.99)	5.06 (3.43 to 7.46)	0.96 (0.94 to 0.98)	0.02 (0.02 to 0.03)	0.99 (0.99 to 0.99)	2.62 (1.86 to 3.70)	0.99 (0.98 to 0.99)
PEWS	0.12 (0.09 to 0.14)	0.95 (0.95 to 0.95)	2.38 (1.89 to 2.99)	0.93 (0.90 to 0.96)	0.07 (0.06 to 0.07)	0.95 (0.95 to 0.96)	1.43 (1.22 to 1.68)	0.98 (0.97 to 0.99)
**Predictive value for tachycardia**								
APLS	0.30 (0.27 to 0.34)	0.85 (0.84 to 0.86)	2.02 (1.78 to 2.29)	0.82 (0.78 to 0.87)	0.24 (0.23 to 0.26)	0.89 (0.88 to 0.89)	2.23 (2.04 to 2.43)	0.85 (0.83 to 0.87)
PALS/sepsis	0.25 (0.22 to 0.28)	0.85 (0.84 to 0.85)	1.60 (1.39 to 1.85)	0.89 (0.85 to 0.93)	0.22 (0.20 to 0.23)	0.87 (0.87 to 0.88)	1.71 (1.57 to 1.87)	0.89 (0.88 to 0.91)
PEWS	0.55 (0.51 to 0.59)	0.63 (0.62 to 0.64)	1.46 (1.36 to 1.58)	0.72 (0.66 to 0.79)	0.50 (0.49 to 0.52)	0.68 (0.67 to 0.69)	1.58 (1.51 to 1.66)	0.73 (0.71 to 0.76)
**Predictive value for tachycardia AND hypotension**								
APLS	0.05 (0.03 to 0.07)	0.99 (0.99 to 0.99)	6.52 (4.26 to 9.96)	0.96 (0.94 to 0.98)	0.02 (0.02 to 0.03)	0.99 (0.99 to 0.99)	6.54 (4.05 to 10.6)	0.98 (0.98 to 0.99)
PALS/sepsis	0.02 (0.01 to 0.04)	0.99 (0.99 to 0.99)	11.9 (6.16 to 23.3)	0.98 (0.97 to 0.99)	0.01 (0.00 to 0.01)	0.99 (0.99 to 0.99)	5.21 (2.35 to 11.6)	0.99 (0.99 to 0.99)
PEWS	0.08 (0.06 to 0.09)	0.98 (0.98 to 0.98)	4.16 (3.06 to 5.66)	0.94 (0.92 to 0.96)	0.04 (0.03 to 0.04)	0.99 (0.98 to 0.99)	3.06 (2.35 to 3.99)	0.97 (0.97 to 0.98)
**Predictive value for tachycardia OR hypotension**								
APLS	0.37 (0.33 to 0.40)	0.81 (0.81 to 0.82)	1.98 (1.77 to 2.21)	0.78 (0.73 to 0.83)	0.28 (0.27 to 0.29)	0.85 (0.85 to 0.86)	1.96 (1.81 to 2.11)	0.84 (0.82 to 0.86)
PALS/sepsis	0.27 (0.24 to 0.31)	0.84 (0.83 to 0.84)	1.69 (1.48 to 1.93)	0.87 (0.83 to 0.91)	0.23 (0.22 to 0.24)	0.87 (0.85 to 0.87)	1.73 (1.59 to 1.88)	0.89 (0.87 to 0.91)
PEWS	0.59 (0.55 to 0.63)	0.59 (0.59 to 0.60)	1.45 (1.35 to 1.56)	0.69 (0.63 to 0.76)	0.53 (0.51 to 0.54)	0.65 (0.64 to 0.66)	1.51 (1.44 to 1.58)	0.73 (0.69 to 0.75)

APLS, Advanced Paediatric Life Support; ICU, intensive care unit; PALS, Paediatric Advanced Life Support; PEWS, Paediatric Early Warning Score.

Average values for Shock Index decreased with age. Stratified by age, Shock Index was associated with ICU admission (range OR 1.07–1.22) and hospitalisation (range OR 1.06–1.19) (table 3). The discriminative ability for Shock Index was poor for admission to ICU (range AUC 0.59–0.63) or admission to the hospital (range AUC 0.58–0.62) (online supplementary appendix 5). The identified cut-offs per age group had low sensitivity (range 0.27–0.42) and moderate specificity (range 0.79–0.91) for ICU admission. None of the identified Shock Index cut-offs had acceptable positive or negative likelihood ratios (online supplementary appendix 6).

The APLS Shock Index cut-off performed similarly with low sensitivity and high specificity (online supplementary appendix 7). The positive likelihood ratio was 3.86 (95% CI 3.1 to 4.8) and negative likelihood ratio was 0.89 (95% CI 0.87 to 0.92).

In febrile children, patients with ill appearance and surgical patients, the hypotension and Shock Index cut-offs showed similar performance. For Shock Index, the highest AUC was found for febrile patients aged >10 years for ICU admission (0.75 95% CI 0.63 to 0.87) (online supplementary appendix 8).

## Discussion

In our observational cohort, hypotension has a significant association with serious illness when corrected for tachycardia. However, hypotension showed low sensitivity and high specificity for serious illness in children with routinely measured BP in the ED. The combination of hypotension and tachycardia did not improve the sensitivity further. In addition, although Shock Index was associated with serious illness, acceptable cut-off values could not be identified for different age groups.

Accurate reference values for abnormal vital signs are essential to avoid misclassification. Values based on healthy children may not be accurate for children in the ED, as ill children may present with pain and distress which influences heart rate and BP values. Expert-based cut-offs for low BP are currently used. However, these are not based on large studies and show large variation and are therefore not a good alternative. For example, more than 50% of the children with hypotension according to the APLS were discharged home following ED visit. Two recent studies presented BP reference ranges and distributions for critically ill children but validated reference values for the paediatric ED population are lacking.^28 29^

Hypotension is considered a late sign of illness that is preceded by an increase in heart rate. To preserve cardiac output, children compensate by elevating heart rate and systemic vascular resistance. When this compensatory mechanism is inadequate, BP could drop which may indicate shock.^7^ Our study showed that heart rate and BP were not correlated. In particular, high z-scores of heart rate did not correlate with low z-scores of BP. Moreover, irrespective of tachycardia, cut-offs for hypotension showed a significant association with serious illness.

We focused on tachycardia as this is an early indicator of critical illness and these children could benefit from measuring BP. Bradycardia, however, indicates irreversible shock. Seriously ill children with bradycardia present with lack of perfusion resulting in cardiopulmonary arrest.^30^ Therefore, BP measurement could have limited additional value in children with bradycardia. Furthermore, we did not analyse other predictors of serious illness. In practice, however, heart rate and BP are evaluated with other clinical markers which can be more sensitive predictors for serious illness. Future studies should focus on the combination of BP and other clinical predictors to evaluate the additional value of BP in practice.

Shock Index is associated with mortality in children with septic shock.^12 13^ Research on Shock Index in EDs has mainly focused on injured patients.^14 15^ No reference values exist for the whole age range in children. Acker *et al* proposed age-adjusted cut-offs according to normal vital signs for children >4 years. However, a recent study showed that 2.3% of healthy children had abnormal values according to this definition.^6^ Our study found an association between Shock Index and serious illness in different age groups. For children >12 years a 0.1 unit increase in Shock Index relates to odds of 1.22 for ICU admission. However, the discriminative ability for Shock Index was poor. In general, neither of the identified cut-off values had both acceptable sensitivity and specificity.

We focused our analysis on high Shock Index values to detect severe illness. We acknowledge that low Shock Index values are also abnormal. Due to the vasopressor response, patients with increased intracranial pressure will have low heart rate and high BP leading to low Shock Index values.

Although hypotension showed high specificity for serious illness, the sensitivity was very low, regardless of the used definition. The combination of hypotension and tachycardia did not improve the sensitivity or the specificity for predicting serious illness. PALS^19^ had good rule-in value having good specificity and high positive likelihood ratios. However, for early recognition of severely ill children in the ED, it is important to rule out serious illness. Hypotension and tachycardia lack these characteristics, having low sensitivity and poor negative likelihood ratios for serious illness. Considering that accurate BP measurement is time-consuming for nurses,^31^ these results suggest limited value of routine BP measurement in all children attending the ED.

Strengths of this study are the use of three hypotension cut-offs that are widely used in clinical practice. In addition, our analyses were based on a large cohort of paediatric ED patients of all ages with different presenting problems. We used routine data and therefore our results are representative of clinical practice.

This study has some limitations. First, patients were included when BP and heart rate were measured. This selected group is more severely ill, comprising older children, more highly urgent cases and more ICU admissions. This could potentially bias our findings. However, this reflects measurement of BP in the practice of the ED. The frequency of BP investigation and the increase with age and urgency was similar to previous studies.^1 2^ In addition, the population of our tertiary university hospital consists of more children with comorbidities and more severely ill children. In settings with low prevalence of serious illness, less yield could be expected. Second, we used hospital admission and ICU admission to define serious illness. These outcomes are widely used in literature and are applicable to large data sets.^21 32 33^ As reasons for ICU admission following ED visit include life-threatening conditions, the presence of hypotension could have influenced the decision for ICU admission. Hospital admission could occur for various conditions as fractures or bronchiolitis which are unlikely to develop low BP. Furthermore, accurate measurement of BP in children in the ED is challenging. Movement of limbs and uncooperativeness interfere with the measurements. Moreover, the correct cuff size and technique need to be applied. Therefore, the quality of BP measurement should be taken into account.

Finally, our study aimed to evaluate the value of routine BP measurements in children for the recognition of serious illness. We acknowledge that BP measurement may be indicated in the ED for diagnostics, detection of hypertension, follow-up or therapy monitoring.

## Conclusion

Our observational study demonstrates that hypotension is associated with serious illness, independent of heart rate. Although the specificity of hypotension is high, the sensitivity for serious illness is very low. The combination of hypotension and tachycardia did not further improve the sensitivity. Shock Index is related to serious illness, however we could not identify acceptable cut-off values. These findings suggest limited value of measuring routine BP to detect hypotension in all attending children. Future studies need to investigate which specific patients could benefit from BP measurement and should focus on developing accurate reference values for hypotension and Shock Index that are applicable in the ED.
