# Supplementary material for: Association between hypotension and serious illness in the emergency department: an observational study
Source: Arch Dis Child. 2019 Apr 4;105(6):545–51. doi: 10.1136/archdischild-2018-316231 (PMC7285787; doi:10.1136/archdischild-2018-316231)
Supplement: Supplementary data [file archdischild-2018-316231supp001.pdf]

Supplemental information

Appendix 1 – Definitions of tachycardia

Table 1 Definitions of tachycardia per different age groups in heart rate/minute

**APLS**(1)	
0-3 m	>170
3 m-1.5 y	>160
1.5 y-2 y	>155
2-3 y	>150
3-4 y	>140
4-6 y	>135
6-8 y	>130
8-12 y	>120
12-14 y	>115
>14 y	>110
**PEWS** (2)	
0 -3 m	≥ 150
3- 12 m	≥ 150
1- 4 y	≥ 120
4-12 y	≥ 110
>12 y	≥ 100
**Septic shock identification tool**(3)	
0- 3 m	>205
≥ 3m - 2 y	>190
≥ 2 - 10 y	>140
≥ 10 - 13 y	>100
> 13y	>100

m, month; y, year

Appendix 2 – Definition of high shock index APLS

Table 2 Cut off high shock index according to APLS (hypotension value/tachycardia value)

<4 weeks	2.27
4 weeks - 6 weeks	2.27
6 weeks - 3 months	2.27
3-6 months	2.13
6-12 months	2.13
1-1.5 years	2.13
1.5-2 years	2.07
2-3 years	1.88
3-4 years	1.75
4-5 years	1.69
5-6 years	1.50
6-7 years	1.44
7-8 years	1.44
8-10 years	1.33
10-12 years	1.28
12-13 years	1.10
13-14 years	1.10
14-16 years	1.05

Appendix 3 – Characteristics patients with no blood pressure/heart rate measurement

Table 3 Characteristics of patients without blood pressure and heart rate measurement

	**N=34,763**
**Male; n %**	20,444 (58.8)
**Age in years; median, (IQR)**	3.40 (1.16-8.45)
**Age category; n (%)**	
0 - 1 year	7799 (22.4)
1 - 2 years	5067 (14.6)
2 - 5 years	8057 (23.2)
5 - 12 years	9394 (27.0)
12 - 16 years	4446 (12.8)
**MTS urgency; n (%)**	
Emergent / Very urgent	3828 (11.0)
Urgent	14847 (42.7)
Standard/ Non urgent	14786 (42.5)
Missing	1299 (3.7)
**Disposition; n (%)**	
Admission general ward	5572 (16.0)
Intensive care	501 (1.4)
Died	- [1]
Discharge	28194 (81.1)
Other	340 (1.0)
Missing	156 (0.4)

IQR, interquartile range; MTS, Manchester Triage System

[1]Children who died were excluded

Appendix 4 – Frequencies of hypotension and tachycardia

Table 4 Frequencies of hypotension and tachycardia according to 3 different cut-offs (N=10,698)

**Hypotension; n (%)**	
APLS	504 (4.7)
PALS/septic shock screening tool	133 (1.2)
PEWS by Parshuram	571 (5.3)
**Tachycardia; n (%)**	
APLS	1692 (15.8)
PALS/septic shock screening tool	1709 (18.0)
PEWS by Parshuram	4113 (38.4)

Appendix 5 – Area under the curve (AUC) for the reveiver operating characteristics with 95% CI for shock index

Table 5 Area under the curve (AUC) for the receiver operating characteristics with 95% CI for shock index

	ICU admission	Hospital admission
All ages	0.63 (0.60-0.65)	0.63 (0.60-0.65)
0-2 years (n=1588)	0.63 (0.58-0.67)	0.62 (0.59-0.64)
2-10 years (n=5011)	0.56 (0.52-0.60)	0.58 (0.56-0.59)
10-16 years (n=4099)	0.59 (0.54-0.64)	0.58 (0.56-0.59)

CI, confidence interval; ICU, Intensive care unit

Appendix 6 – Predictive value for high cut-offs of shock index

Table 6 Predictive value for cut-offs of shock index according to different age groups

**ICU admission**	Shock index Cut-off*	Sensitivity (95% CI)	Specificity (95% CI)	Positive likelihood ratio (95% CI)	Negative likelihood ratio (95% CI)
0-2 years (n=1588)	1.63	0.42 (0.35-0.49)	0.79 (0.77-0.81)	2.00 (1.65-2.44)	0.74 (0.65-0.83)
2-10 years (n=5011)	1.18	0.36 (0.79-0.81)	0.80 (0.79-0.81)	1.83 (1.53-2.18)	0.79 (0.72-0.88)
10-16 years (n=4099)	1.05	0.27 (0.21-0.34)	0.91 (0.91-0.92)	3.16 (2.45-4.07)	0.79 (0.73-0.87)
**Hospital admission**					
0-2 years (n=1588)	1.45	0.48 (0.45-0.51)	0.72 (0.69-0.76)	1.74 (1.52-1.99)	0.72 (0.67-0.78)
2-10 years (n=5011)	1.13	0.34 (0.32-0.37)	0.79 (0.77-0.80)	1.63 (1.49-1.79)	0.83 (0.80-0.86)
10-16 years (n=4099)	0.91	0.30 (0.28-0.33)	0.82 (0.81-0.84)	1.73 (1.54-1.94)	0.84 (0.81-0.88)

CI, confidence interval; ICU, intensive care unit
*Cut-off value determined by Youden’s index

Appendix 7 Predictive value of high shock index: cut-off defined by APLS abnormal vital signs

Table 7 Predictive value of high shock index cut-off by APLS*

**ICU admission**	Sensitivity (95% CI)	Specificity (95% CI)	Positive likelihood ratio (95% CI)	Negative likelihood ratio (95% CI)
Total	0.14 (0.11-0.17)	0.96 (0.96-0.97)	3.86 (3.1-4.8)	0.89 (0.87-0.92)
Ill appearance	0.22 (0.17-0.28)	0.92 (0.90-0.93)	2.71 (1.95-3.75)	0.85 (0.79-0.92)
Fever	0.20 (0.14-0.28)	0.92 (0.91-0.93)	2.49 (1.71-3.65)	0.87 (0.79-0.95)
Surgical	0.04 (0.02-0.08)	0.99 (0.99-0.00)	4.80 (1.74-13.3)	0.97 (0.94-1.0)
**Hospital admission**				
Total	0.08 (0.07-0.08)	0.98 (0.97-0.98)	3.33 (2.75-4.03)	0.95 (0.94-0.96)
Ill appearance	0.13 (0.11-0.15)	0.96 (0.94-0.97)	3.03 (1.95-4.72)	0.91 (0.88-0.94)
Fever	0.15 (0.13-0.17)	0.95 (0.94-0.96)	3.23 (2.48-4.22)	0.89 (0.87-0.92)
Surgical	0.02 (0.01-0.03)	0.99 (0.99-0.99)	2.79 (1.05-7.41)	0.99 (0.98-1)

CI, confidence interval; ICU, intensive care unit

*APLS cut-off calculated by dividing APLS tachycardia value to APLS hypotension value for each age group

Appendix 8 Results of subgroup analysis in children with fever, ill appearance, surgical.

Table 8 Predictive value (95% CI) for hypotension and/or tachycardia for ICU admission for subgroups: fever, ill appearance, surgical

	**Fever** (n=2,678)				**Ill appearance** (n=1,436)				**Surgical** (n=1,912)			
	**Sens (95% CI)**	**Spec (95% CI)**	**Positive likelihood ratio (95% CI)**	**Negative likelihood ratio (95% CI)**	**Sens (95% CI)**	**Spec (95% CI)**	**Positive likelihood ratio (95% CI)**	**Negative likelihood ratio (95% CI)**	**Sens (95% CI)**	**Spec (95% CI)**	**Positive likelihood ratio (95% CI)**	**Negative likelihood ratio (95% CI)**
**Predictive value for different cut-offs for hypotension**												
APLS	0.11 (0.06-0.18)	0.97 (0.96-0.97)	3.19 (1.83-5.54)	0.92 (0.87-0.98)	0.13 (0.09-0.18)	0.95 (0.94-0.96)	2.78 (1.78-4.33)	0.91 (0.86-0.97)	0.09 (0.05-0.14)	0.98 (0.97-0.98)	3.94 (2.11-7.38)	0.94 (0.89-0.98)
PALS / sepsis	0.08 (0.04-0.14)	0.99 (0.99-0.99)	5.35 (2.53-11.3)	0.94 (0.89-0.99)	0.08 (0.05-0.12)	0.99 (0.98-0.99)	6.04 (3.04-12.0)	0.93 (0.89-0.97)	0.04 (0.02-0.08)	0.99 (0.99-0.99)	7.81 (2.59-23.5)	0.97 (0.94-1)
PEWS	0.13 (0.08-0.21)	0.95 (0.95-0.96)	2.89 (1.77-4.71)	0.91 (0.85-0.98)	0.15 (0.11-0.21)	0.94 (0.92-0.95)	2.43 (1.63-3.62)	0.91 (0.85-0.96)	0.09 (0.06-0.16)	0.97 (0.96-0.98)	3.18 (1.81-5.57)	0.93 (0.88-0.98)
**Predictive value for different cut-offs for tachycardia**												
APLS	0.56 (0.47-0.64)	0.63 (0.61-0.65)	1.49 (1.27-1.77)	0.70 (0.57-0.86)	0.44 (0.37-0.51)	0.64 (0.61-0.67)	1.23 (1.03-1.46)	0.87 (0.77-0.99)	0.12 (0.08-0.18)	0.96 (0.95-0.97)	2.91 (1.77-4.79)	0.92 (0.86-0.98)
PALS / sepsis	0.41 (0.32-0.49)	0.69 (0.69-0.70)	1.30 (1.04-1.63)	0.86 (0.74-1.00)	0.32 (0.25-0.38)	0.69 (0.66-0.71)	1.01 (0.81-1.26)	0.99 (0.89-1.10)	0.13 (0.09-0.20)	0.99 (0.99-0.99)	20.3 (9.88-41.9)	0.87 (0.82-0.93)
PEWS	0.86 (0.78-0.91)	0.28 (0.26-0.29)	1.19 (1.11-1.29)	0.51 (0.32-0.79)	0.70 (0.64-0.76)	0.34 (0.31-0.36)	1.06 (0.96-1.17)	0.88 (0.69-1.11)	0.33 (0.25-0.41)	0.80 (0.78-0.82)	1.65 (1.28-2.13)	0.84 (0.75-0.95)
**Predictive value for different cut-offs for tachycardia AND hypotension**												
APLS	0.08 (0.04-0.14)	0.98 (0.98-0.99)	4.57 (2.27-9.16)	0.94 (0.89-0.99)	0.09 (0.06-0.14)	0.98 (0.97-0.99)	4.76 (2.59-8.75)	0.93 (0.89-0.97)	NA*	NA*	NA*	NA*
PALS / sepsis	0.04 (0.02-0.09)	0.99 (0.99-0.99)	9.69 (3.42-27.4)	0.96 (0.93-0.99)	0.03 (0.01-0.07)	0.99 (0.990.99)	4.29 (1.55-11.9)	0.98 (0.95-1.00)	NA*	NA*	NA*	NA*
PEWS	0.12 (0.07-0.19)	0.97 (0.96-0.97)	3.39 (1.99-5.78)	0.92 (0.86-0.98)	0.12 (0.08-0.17)	0.96 (0.95-0.97)	3.09 (1.92-4.95)	0.92 (0.87-0.97)	0.05 (0.02-0.09)	0.99 (0.99-0.99)	6.73 (2.73-16.6)	0.96 (0.92-0.99)
**Predictive value for different cut-offs for tachycardia OR hypotension**												
APLS	0.59 (0.50-0.68)	0.61 (0.59-0.63)	1.52 (1.29-1.77)	0.67 (0.54-0.83)	0.48 (0.41-0.55)	0.61 (0.49-0.64)	1.25 (1.06-1.46)	0.85 (0.73-0.98)	0.18 (0.12-0.25)	0.94 (0.93-0.95)	2.89 (1.94-4.31)	0.88 (0.81-0.95)
PALS / sepsis	0.44 (0.36-0.53)	0.68 (0.66-0.69)	1.39 (1.13-1.71)	0.82 (0.69-0.96)	0.36 (0.29-0.43)	0.68 (0.66-0.71)	1.14 (0.93-1.39)	0.94 (0.84-1.05)	0.15 (0.09-0.22)	0.93 (0.92-0.94)	2.24 (1.46-3.45)	0.91 (0.85-0.98)
PEWS	0.88 (0.80-0.92)	0.27 (0.25-0.29)	1.19 (1.11-1.29)	0.47 (0.29-0.75)	0.74 (0.67-0.79)	0.31 (0.29-0.34)	1.07 (0.98-1.18)	0.84 (0.67-1.08)	0.38 (0.30-0.46)	0.78 (0.76-0.79)	1.69 (1.34-2.13)	0.80 (0.70-0.91)

*<5 cases in 2x2 table

CI, confidence interval; ICU, intensive care unit; NA, not applicable; sens, sensitivity; spec, specificity

Table 9 Predictive value for hypotension and/or tachycardia for hospital admission for subgroups: fever, ill appearance, surgical

	**Fever** (n=2,678)				**Ill appearance** (n=1,436)				**Surgical** (n=1,912)			
	**Sens (95% CI)**	**Spec (95% CI)**	**Positive likelihood ratio (95% CI)**	**Negative likelihood ratio (95% CI)**	**Sens (95% CI)**	**Spec (95% CI)**	**Positive likelihood ratio (95% CI)**	**Negative likelihood ratio (95% CI)**	**Sens (95% CI)**	**Spec (95% CI)**	**Positive likelihood ratio (95% CI)**	**Negative likelihood ratio (95% CI)**
**Predictive value for hypotension**												
APLS	0.06 (0.05-0.08)	0.98 (0.97-0.98)	2.68 (1.8-3.99)	0.96 (0.95-0.98)	0.07 (0.06-0.09)	0.97 (0.95-0.98)	2.16 (1.28-3.64)	0.96 (0.94-0.98)	0.03 (0.02-0.04)	0.97 (0.96-0.98)	1.09 (0.63-1.90)	0.99 (0.98-1.01)
PALS / sepsis	0.02 (0.02-0.04)	0.99 (0.99-0.99)	5.63 (2.44-12.9)	0.98 (0.97-0.99)	NA*	NA*	NA*	NA*	NA*	NA*	NA*	NA*
PEWS	0.07 (0.06-0.09)	0.96 (0.95-0.97)	1.89 (1.35-2.62)	0.97 (0.95-0.99)	0.09 (0.07-0.11)	0.95 (0.93-0.97)	1.89 (1.22-2.95)	0.96 (0.93-0.98)	0.05 (0.04-0.07)	0.97 (0.96-0.98)	1.81 (1.14-2.89)	0.98 (0.96-0.99)
**Predictive value for tachycardia**												
APLS	0.52 (0.49-0.55)	0.71 (0.69-0.73)	1.79 (1.63-1.97)	0.67 (0.63-0.72)	0.42 (0.39-0.45)	0.73 (0.68-0.76)	1.54 (1.31-1.80)	0.79 (0.74-0.86)	0.07 (0.06-0.09)	0.97 (0.96-0.98)	2.53 (1.66-3.86)	0.96 (0.93-0.98)
PALS / sepsis	0.41 (0.38-0.44)	0.74 (0.72-0.76)	1.59 (1.43-1.77)	0.79 (0.75-0.84)	0.35 (0.32-0.38)	0.75 (0.71-0.78)	1.37 (1.15-1.63)	0.88 (0.82-0.94)	0.09 (0.07-0.11)	0.95 (0.93-0.96)	1.73 (1.24-2.42)	0.96 (0.94-0.99)
PEWS	0.81 (0.78-0.83)	0.33 (0.30-0.35)	1.19 (1.14-1.25)	0.59 (0.52-0.69)	0.69 (0.67-0.73)	0.38 (0.34-0.42)	1.13 (1.04-1.22)	0.79 (0.68-0.92)	0.28 (0.25-0.31)	0.85 (0.82-0.87)	1.83 (1.53-2.18)	0.85 (0.81-0.89)
**Predictive value for tachycardia AND hypotension**												
APLS	0.04 (0.03-0.06)	0.99 (0.99-0.99)	9.90 (4.48-21.9)	0.96 (0.95-0.98)	NA*	NA*	NA*	NA*	NA*	NA*	NA*	NA*
PALS / sepsis	NA*	NA*	NA*	NA*	NA*	NA*	NA*	NA*	NA*	NA*	NA*	NA*
PEWS	0.06 (0.05-0.08)	0.98 (0.97-0.98)	2.65 (1.79-3.93)	0.96 (0.94-0.98)	0.07 (0.05-0.08)	0.98 (0.96-0.99)	3.39 (1.76-6.57)	0.98 (0.96-0.99)	0.02 (0.01-0.03)	0.99 (0.99-0.99)	2.59 (1.04-6.47)	0.99 (0.98-1.00)
**Predictive value for tachycardia OR hypotension**												
APLS	0.54 (0.51-0.57)	0.69 (0.67-0.71)	1.65 (1.51-1.79)	0.66 (0.61-0.72)	0.45 (0.42-0.49)	0.69 (0.65-0.73)	1.49 (1.28-1.73)	0.79 (0.72-0.85)	0.09 (0.07-0.12)	0.95 (0.93-0.96)	1.75 (1.26-2.43)	0.96 (0.93-0.98)
PALS / sepsis	0.42 (0.39-0.45)	0.74 (0.72-0.76)	1.60 (1.44-1.78)	0.79 (0.74-0.83)	0.36 (0.33-0.39)	0.74 (0.70-0.78)	1.39 (1.17-1.65)	0.86 (0.80 - 0.93)	0.09 (0.08-0.12)	0.95 (0.93-0.96)	1.86 (1.35-2.59)	0.95 (0.93-0.98)
PEWS	0.81 (0.79-0.84)	0.31 (0.29-0.33)	1.18 (1.13-1.23)	0.59 (0.52-0.69)	0.72 (0.69-0.75)	0.35 (0.31-0.39)	1.12 (1.04-1.20)	0.79 (0.67 - 0.92)	0.31 (0.28-0.35)	0.82 (0.80-0.85)	1.79 (1.53-2.12)	0.83 (0.78-0.88)

*<5 cases available in 2x2 table.

CI, confidence interval; NA, not applicable; sens, sensitivity; spec, specificity

Table 10 Predictive value of shock index for febrile patients (n=2620)

	AUC (95% CI)	Shock index cut-off*	Sensitivity (95% CI)	Specificity (95% CI)	Positive likelihood ratio (95% CI)	Negative likelihood ratio (95% CI)
**ICU admission**						
0-2y (n=520)	0.59 (0.49-0.69)	1.54	0.59 (0.44-0.72)	0.64 (0.59-0.68)	1.63 (1.24-2.13)	0.65 (0.46-0.92)
2-10y (n=1497)	0.59 (0.52-0.68)	1.19	0.62 (0.49-0.73)	0.62 (0.59-0.64)	1.62 (1.31-2.02)	0.62 (0.44-0.87)
10-16y (n=661)	0.75 (0.63-0.87)	1.08	0.74 (0.51-0.88)	0.75 (0.71-0.78)	2.92 (2.16-3.94)	0.35 (0.17-0.75)
**Hospital admission**						
0-2y	0.67 (0.62-0.72)	1.47	0.60 (0.54-0.66)	0.68 (0.62-0.73)	1.88 (1.53-2.31)	0.59 (0.49-0.69)
2-10y	0.61 (0.58-0.64)	1.16	0.57 (0.53-0.61)	0.63 (0.59-0.66)	1.53 (1.37-1.71)	0.69 (0.61-0.77)
10-16y	0.63 (0.59-0.68)	0.95	0.59 (0.54-0.66)	0.61 (0.56-0.66)	1.53 (1.31-1.79)	0.66 (0.56-0.78)

AUC, area under the curve; CI, confidence interval; ICU, intensive care unit
*Cut-off value determined by Youden’s index

Table 11 Predictive value of shock index for ill appearance (n=1436)

	AUC	Shock index cut-off*	Sensitivity (95% CI)	Specificity (95% CI)	Positive likelihood ratio (95% CI)	Negative likelihood ratio (95% CI)
**ICU admission**						
0-2y (n=337)	0.56 (0.49-0.64)	1.90	0.25 (0.17-0.35)	0.88 (0.84-0.92)	2.08 (1.26-3.42)	0.86 (0.85-0.97)
2-10y (n=682)	0.49 (0.40-0.57)	1.64	0.13 (0.07-0.24)	0.95 (0.94-0.97)	2.91 (1.39-6.09)	0.91 (0.82-1.01)
10-16y (n=417)	0.60 (0.50-0.70)	1.06	0.45 (0.31-0.59)	0.79 (0.74-0.83)	2.09 (1.44-3.04)	0.70 (0.54-0.91)
**Hospital admission**						
0-2y	0.67 (0.61-0.73)	1.49	0.55 (0.49-0.61)	0.76 (0.65-0.84)	2.25 (1.48-3.41)	0.59 (0.49-0.72)
2-10y	0.52 (0.48-0.56)	1.15	0.50 (0.45-0.55)	0.58 (0.52-0.63)	1.18 (0.99-1.40)	0.87 (0.75-0.99)
10-16y	0.57 (0.51-0.62)	1.06	0.31 (0.25-0.37)	0.86 (0.80-0.91)	2.24 (1.47-3.41)	0.80 (0.72-0.89)

AUC, area under the curve; CI, confidence interval; ICU, intensive care unit
*Cut-off value determined by Youden’s index

Table 12 Predictive value of shock index for surgical patients (n=1912)

	AUC	Shock index cut-off*	Sensitivity (95% CI)	Specificity (95% CI)	Positive likelihood ratio (95% CI)	Negative likelihood ratio (95% CI)
**ICU admission**						
0-2y (n=230)	0.54 (0.39-0.69)	1.41	0.24 (0.11-0.45)	0.89 (0.85-0.93)	2.37 (0.99-5.63)	0.85 (0.66-1.08)
2-10y (n=965)	0.51 (0.43-0.59)	1.18	0.19 (0.11-0.29)	0.93 (0.91-0.95)	2.78 (1.61-4.81)	0.87 (0.78-0.98)
10-16y (n=717)	0.55 (0.46-0.64)	0.80	0.37 (0.25-0.51)	0.76 (0.73-0.79)	1.56 (1.07-2.28)	0.82 (0.66-1.02)
**Hospital admission**						
0-2y	0.49 (0.42-0.57)	0.95	0.85 (0.77-0.90)	0.21 (0.14-0.29)	1.07 (0.95-1.21)	0.73 (0.42-1.27)
2-10y	0.53 (0.49-0.57)	1.18	0.12 (0.09-0.15)	0.96 (0.94-0.97)	2.78 (1.73-4.48)	0.92 (0.88-0.96)
10-16y	0.54 (0.49-0.59)	0.80	0.29 (0.24-0.36)	0.78 (0.73-0.81)	1.33 (1.03-1.72)	0.91 (0.83-0.99)

AUC, area under the curve; CI, confidence interval; ICU, intensive care unit
*Cut-off value determined by Youden's index

References

1. Turner NM, Leroy PL. Advanced paediatric life support. 5th ed: Bohn Stafleu van Loghum; 2017.
2. Parshuram CS, Duncan HP, Joffe AR*, et al.* Multicentre validation of the bedside paediatric early warning system score: a severity of illness score to detect evolving critical illness in hospitalised children. *Crit Care*. 2011;**15**:R184 doi: cc10337 [pii]

10.1186/cc10337 [published Online First: 2011/08/05].
3. Davis AL, Carcillo JA, Aneja RK*, et al.* American College of Critical Care Medicine Clinical Practice Parameters for Hemodynamic Support of Pediatric and Neonatal Septic Shock. *Critical Care Medicine*. 2017;**45**:1061-93 doi: 10.1097/ccm.0000000000002425 [published Online.
